# Early weaning increases aggression and stereotypic behaviour in cats

**DOI:** 10.1038/s41598-017-11173-5

**Published:** 2017-09-04

**Authors:** Milla K. Ahola, Katariina Vapalahti, Hannes Lohi

**Affiliations:** 1 Department of Veterinary Biosciences, University of Helsinki, 00014 Helsinki, Finland; 2 Research Programs Unit, Molecular Neurology, University of Helsinki, 00014 Helsinki, Finland; 3 The Folkhälsan Institute of Genetics, 00290 Helsinki, Finland; 4 Section of Ecology, Department of Biology, University of Turku, FI-20014 Turku, Finland

## Abstract

Behaviour problems are common in companion felines, and problematic behaviour may be a sign of chronic stress. In laboratory animals, early weaning increases the risk for aggression, anxiety, and stereotypic behaviour. However, very few studies have focused on early weaning in one of the world’s most popular pets, the domestic cat, although weaning soon after the critical period of socialisation is common practice. To study the effects of early weaning (<12 weeks) on behaviour, a large data set (N = 5726, 40 breeds) was collected from home-living domestic cats through a questionnaire survey. The results show that weaning before 8 weeks of age increases the risk for aggression, but not fearful behaviour. Moreover, cats weaned after 14 weeks of age have a lower probability for aggression towards strangers than early weaned cats and a lower probability for stereotypic behaviour (excessive grooming) than cats weaned at 12 weeks. The effect of weaning age on stereotypic behaviour is partially explained by the effects on aggression. These findings indicate that early weaning has a detrimental effect on behaviour, and suggest delayed weaning as a simple and inexpensive approach to significantly improve the welfare of millions of domestic cats.

## Introduction

Early weaning, defined as permanent separation from the mother before the time it would occur in nature^1^, has several impacts on health^2–5^ and behaviour of animals. Early weaning may lead to neurobiological changes, for example, alter the neuroendocrine stress response^6^, but this effect has not been found in all studies^7^. Furthermore, early weaning can impair memory^8^ and cognition, such as extinction learning^9^, and it seems to especially blunt social learning^10^. Behavioural changes linked to early weaning are extensive: early weaning may lead to impairments in social behaviour as well as increased anxiety^11, 12^ and aggression^7, 12^, and these behavioural changes may persist for a long time^13^. However, moderate levels of early life stress may, on the other hand, increase resilience towards stressors, displayed by diminished behavioural signs of anxiety and levels of blood cortisol^14^, and the behavioural changes caused by early life stress may be adaptive^15^. Furthermore, many factors may influence the impact of early weaning. For example, the effects of early life stress and early weaning can differ between sexes^13, 16–18^, and the impacts of early weaning may arise only when combined with post-weaning social isolation^18^. Importantly, the effects of early life stress depend on the developmental timing of the stressor^19, 20^ and therefore early weaning after the critical period of socialisation may not induce behavioural changes.

Besides affecting normal behavioural repertoire, early weaning may impact the expression of abnormal behaviours, such as stereotypies^1^, which may be caused by central nervous system dysfunction^21^. Stereotypies are invariable and repeated behaviours that are often expressed when animals experience adverse or frustrating situations^22^. Examples of stereotypic behaviour patterns include rocking and hair plucking in rhesus macaques^23^, crib-biting in horses^24^, and pacing in tigers and other carnivores^25^. Stereotypies are commonly seen in captive animals, both wild and domesticated, but seem to be absent in nature^22^. Many environmental and genetic factors may induce the development of stereotypies. The occurrence of stereotypies is, at least to some extent, heritable^26^ and it may be correlated with personality^27–30^. Furthermore, early weaning has been shown to increase stereotypic behaviour in captive and laboratory animals^1^, for example, tail-biting in mink^31^ and wire-gnawing in mice^32^. In domestic dog, early weaning and poor maternal care has been associated with stereotypic tail chasing^30^. However, early weaning does not necessarily produce stereotypic animals, especially when combined with environmental enrichment^33^. Furthermore, sometimes the increase in stereotypic behaviour can be temporary^7^.

One companion animal in which early weaning is very common is the domestic cat (*Felis catus* Linnaeus 1758), which may be the most popular companion animal in the world, with close to 100 million domestic cats living in Europe alone^34^. Feral cats wean their kittens at four to eight weeks of age, but kittens usually stay with their mother for the first four months of their lives^35^. In cats, the critical period of socialisation occurs between 2 to 8 weeks of age^36^. Consequently, separation from the mother and littermates at an age of eight weeks is common. For example, The Royal Society for the Prevention of Cruelty to Animals^37^ recommends a minimum separation age of eight weeks. Moreover, The American Veterinary Medical Association considers 7–9 weeks of age to be the ideal time to move to a new home^38^, but the effects of weaning at this life stage have not been evaluated. In a previous study, laboratory kittens reared in isolation in a brooder showed deficits in social behaviour, behaved anxiously, and showed difficulties in habituation to novel objects, displayed aggression towards other kittens and showed more random movement than kittens reared with the mother and littermates^39^. Similarly, laboratory cats separated from their mother and siblings at two weeks of age behaved anxiously in novel environments, showed aggression towards other cats and people, and displayed random movement^40^. However, laboratory cats separated at 6 weeks of age did not display higher levels of aggression or random movement than cats weaned at 12 weeks of age^40^, indicating that, at least in laboratory settings, the timing of weaning is important. Stereotypic behaviour in cats is proposed to be in part caused or worsened by early weaning^1^. In a recent study, early weaning was indeed correlated with stereotypic wool sucking in Birman cats^41^.

The aim of this study was to examine the effects of early weaning on the behaviour of domestic cats using the large population-based survey data from our feline health and behaviour questionnaire^42^. Here, we have focused on the effects of early weaning by examining the behavioural differences between early and late weaned conspecifics in a sample of 5726 home-living domestic cats in 40 breeds using logistic regression. In our study, cats separated from the mother before 12 weeks of age are considered early weaned, following the recommendation of The Humane Society of the United States^43^. Accordingly, we considered late weaned cats to be weaned after this 12-week recommended age, with our recommended weaning age group consisting of cats weaned at 12–13 weeks of age and late weaned at 14 weeks of age or older. As early weaning is common in farm and companion animals^1^, research on the topic is important since it may identify possible neural mechanisms and intervention approaches that could significantly improve the welfare and health of millions of animals, including cats, which are popular pets^34^.

## Results

Using logistic regression, we studied the effects of early weaning on behaviour in 5726 home-living domestic cats in 40 breeds. Our study revealed that behavioural problems are common in cats^42^. For example, 41% of cats were at least slightly aggressive towards other cats and nearly 32% displayed at least one bout of wool sucking during their life. We found that several environmental factors affected behaviour (Table 1; Supplementary Table S1; Supplementary Figures S1–S37). We also discovered that our behavioural traits (besides stereotypic behaviour) grouped into three personality factors: extraversion, aggression, and shyness, and these personality factors influenced behaviour. In this study, we focused on the effects of early weaning. As personality factors may influence the effect of weaning age on behaviour, we first ran all the models without any personality traits and then with the relevant personality traits (as defined by model selection) included. We report the former models when the removal of personality traits alters the effect of early weaning significantly.

**Table 1:** Results of logistic regression analyses on the association between different environmental factors and the response variables. P values are controlled for false discovery rate. N = 5726 (personality trait analyses), N = 4925 (wool sucking analysis), N = 5683 (excessive grooming analysis), N = 5550 (owner-evaluated behaviour problem analysis).

Variable	Aggression to family members		Aggression to strangers		Aggression to cats		Shyness to strangers		Contact		DF
	χ^2^	P value	χ^2^	P value	χ^2^	P value	χ^2^	P value	χ^2^	P value	
Weaning age	17.62	**0.042**	26.6	**0.003**	19.02	**0.028**	5.22	0.700	10.64	0.238	7
Sex	0.95	0.466	7.74	**0.021**	14.17	**0.001**	6.37	**0.027**	49.32	**0.0005**	1
Age	0.21	0.749	13.02	**0.002**	63.22	**0.001**	19.04	**0.0004**	42.52	**0.0005**	1
Hormonal status	6.03	**0.042**	5.54	0.057	40.43	**0.001**	49.11	**0.0004**	4.71	0.061	1
Breed	110.96	**0.001**	117.44	**0.001**	104.12	**0.001**	196.65	**0.0004**	146.68	**0.0005**	18
Access to outdoors			25.92	**0.001**	25.94	**0.001**					5
Other cats	69.42	**0.001**	18.47	**0.001**	37.16	**0.001**	4.77	0.057	7.20	**0.019**	1
Shyness			4.93	0.078	27.17	**0.001**			269.71	**0.0005**	1
Extraversion	4.87	**0.073**					78.62	**0.0004**			1
	**Shyness to novel objects**		**Wool sucking**		**Excessive grooming**		**Owner-evaluated behaviour problem**				
**Variable**	**χ** ^2^	**P** **value**	**χ** ^2^	**P** **value**	**χ** ^2^	**P** **value**	**χ** ^2^	**P** **value**	**DF**		
Weaning age	8.39	0.401	24.4	**0.005**	16.08	0.102	18.96	0.071	7		
Sex	0.13	0.783	1.18	0.401	0.12	0.841	0.05	0.897	1		
Age	18.65	**0.0004**	20.29	**0.001**	4.89	0.107	23.34	**0.004**	1		
Hormonal status	63.99	**0.0004**							1		
Breed	171.94	**0.0004**	103.39	**0.001**	43.63	**0.027**	43.24	**0.018**	18		
Access to outdoors	26.57	**0.0004**	57.09	**0.001**	19.49	**0.037**			5		
Other cats	12.47	**0.001**					4.34	0.159	1		
Shyness			39.59	**0.001**	31.96	**0.01**	42.55	**0.004**	1		
Extraversion	42.47	**0.0004**	15.45	**0.001**	5.03	0.105			1		
Aggression			14.52	**0.001**	26.61	**0.01**	129.57	**0.004**	1		
Stereotypic behaviour							43.97	**0.004**	1		

### Effects of early weaning on social behaviour

**Figure 1:**
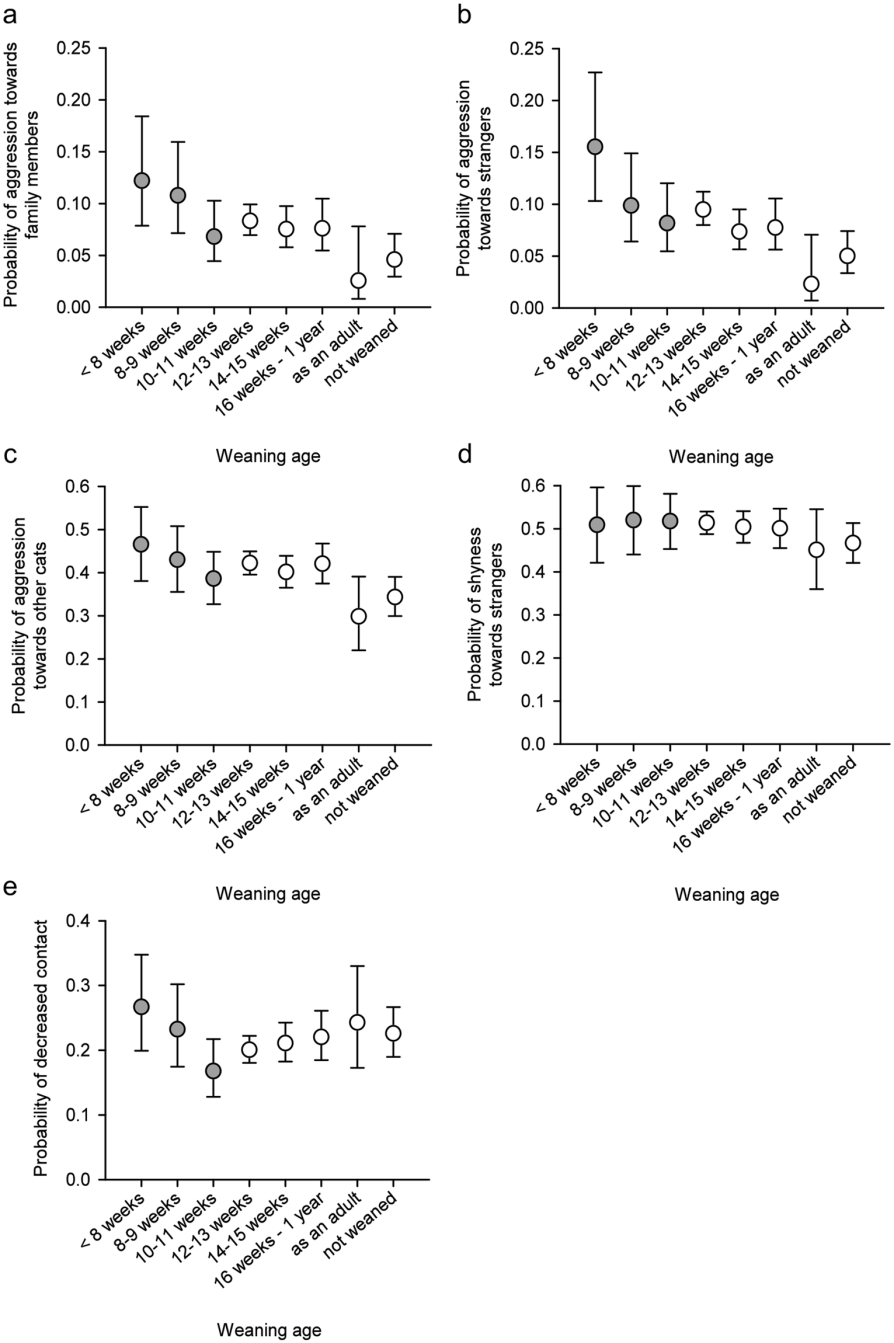
The effect of weaning age on social behaviour in logistic regression analyses. Grey circles are the groups considered early weaned. (**a**) Cats weaned in adulthood were less aggressive towards family members than other cats. (**b**) Cats weaned before 8 weeks of age were more likely aggressive towards strangers than cats weaned at 12–13 weeks of age. Cats weaned at 14–15 weeks of age were less aggressive than early weaned cats. Furthermore, cats weaned in adulthood were less aggressive than other cats. (**c**) Cats weaned in adulthood were less likely to display aggression towards other cats. (**d**) Weaning age did not affect shyness towards strangers. (**e**) Weaning age did not affect probability for decreased contact. Error bars indicate 95% confidence limits. N = 5726.

In the logistic regression analyses (Table 1), cats weaned before 8 weeks of age were significantly more likely to behave aggressively towards strangers than cats weaned at 12–13 weeks of age (Table 2; Fig. 1). Moreover, cats weaned in adulthood or not weaned at all were significantly less likely to show aggression towards other cats, family members, and strangers than other weaning age group cats. Furthermore, cats weaned at 14–15 weeks of age were significantly less likely to display aggression towards strangers than early weaned cats.

**Table 2:** Contrasts between different weaning age groups in logistic regression analyses. DF = 1 in all comparisons. N = 5726 (personality trait analyses), N = 4925 (wool sucking analysis), N = 5683 (excessive grooming analysis), N = 5550 (owner-evaluated behaviour problem analysis).

	<8 weeks vs. 12–13 weeks		8–9 weeks vs. 12–13 weeks		10–11 weeks vs. 12–13 weeks		14–15 weeks vs. 12–13 weeks		14–15 weeks vs. early weaned		Adult/not weaned vs. other groups	
	χ^2^	P	χ^2^	P	χ^2^	P	χ^2^	P	χ^2^	P	χ^2^	P
aggression towards family members	3.00	0.083	1.59	0.207	0.91	0.340	0.52	0.470	1.76	0.184	8.84	**0.003**
aggression towards strangers	5.62	**0.017**	0.04	0.850	0.57	0.452	3.68	0.055	4.43	**0.035**	10.91	**0.001**
aggression towards other cats	0.98	0.323	0.04	0.839	1.31	0.253	1.14	0.287	0.71	0.399	11.52	**0.0007**
shyness towards strangers	0.01	0.914	0.02	0.880	0.01	0.913	0.24	0.622	0.13	0.717	3.04	0.081
contact with people	3.68	0.055	1.06	0.302	1.81	0.178	0.45	0.504	0.12	0.728	0.66	0.416
shyness towards novel objects	0.09	0.762	0.49	0.484	0.003	0.955	0.001	0.972	0.22	0.640	5.20	**0.023**
wool sucking	0.48	0.490	0.83	0.362	0.004	0.949	0.90	0.342	1.85	0.174	13.72	**0.0002**
excessive grooming	0.48	0.488	0.008	0.930	0.00	0.995	6.61	**0.010**	2.61	0.106	3.45	0.063
owner-evaluated behaviour problem	14.07	**0.0002**	0.07	0.798	0.96	0.327	0.74	0.389	5.32	**0.021**	0.21	0.643

### Effects of early weaning on non-social behaviour

**Figure 2:**
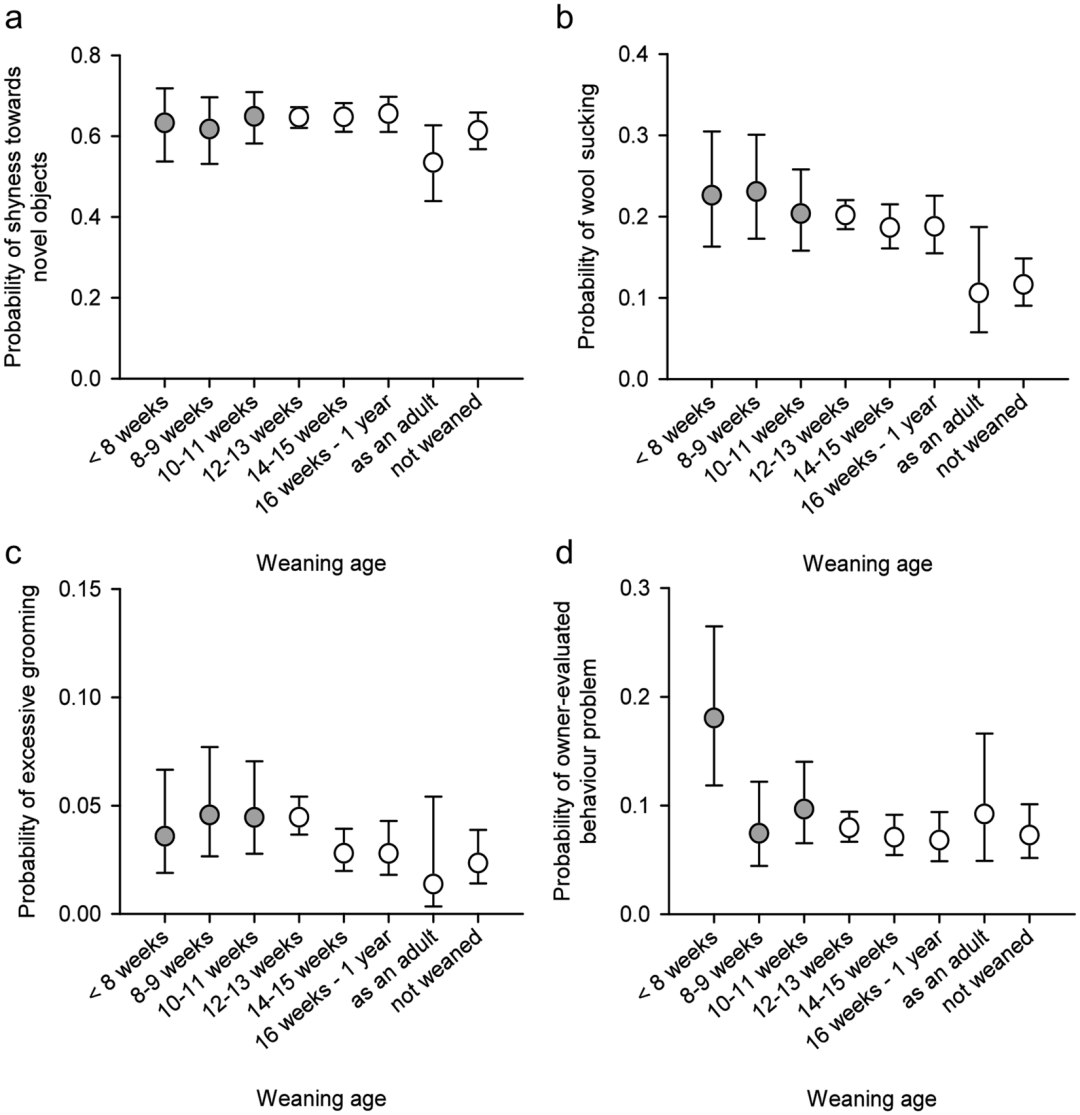
The effect of weaning age on non-social behaviour in logistic regression analyses. Grey circles are the groups considered early weaned. (**a**) Cats weaned in adulthood or not weaned at all had a lower probability for shyness towards novel objects than other cats. (**b**) Cats weaned in adulthood or not weaned at all were less likely to perform wool sucking than other cats. (**c**) Cats weaned at 14–15 weeks of age were less likely to groom excessively than cats weaned 12–13 weeks of age. (**d**) Cats weaned before 8 weeks of age were more likely to have an owner-evaluated behaviour problem than cats weaned at 12–13 weeks of age. Error bars indicate 95% confidence limits. N = 4925 (wool sucking), N = 5683 (excessive grooming), N = 5550 (owner-evaluated behaviour problem), and N = 5726 (shyness).

In logistic regression analysis (Table 1), cats weaned in adulthood or not weaned at all were significantly less likely to display shyness towards novel objects than other cats (Fig. 2; Table 2). Weaning age affected the probability to display stereotypic behaviour as well. When personality traits were left out of the final models, cats weaned at 14–15 weeks of age were less likely to groom excessively than cats weaned at 12–13 weeks of age (χ^2^ = 7.14, DF = 1, P = 0.0075). Cats weaned as adults were less likely to perform stereotypic wool sucking (χ^2^ = 15.19, DF = 1, P < 0.0001) and excessive grooming (χ^2^ = 4.78, DF = 1, P = 0.0288) than other cats. However, the probability of stereotypic behaviour increased with increasing scores in shyness and aggression, and wool sucking increased with increasing extraversion score as well. Despite not detecting significant multicollinearity, when these personality traits were included in the models, as favoured by model selection, the differences between these weaning age groups decreased slightly (Fig. 2; Table 2). The occurrence of stereotypic behaviour seemed to be correlated, as some cats had co-occurring wool sucking and excessive grooming (polychoric r = 0.294, P < 0.001). Cats weaned before 8 weeks of age were significantly more likely to have an owner-evaluated behaviour problem than cats weaned at 12–13 weeks of age (χ^2^ = 18.73, DF = 1, P < 0.0001), but similarly, this probability slightly decreased when including personality traits in the model.

## Discussion

This study showed that early weaning can have detrimental effects on cat behaviour: we found a predisposition for aggression in early weaned cats, and discovered that the late weaned cats were less likely to behave aggressively and display stereotypic behaviour. The lower probability of stereotypic behaviour in the late weaned cats was partially explained by a lower probability of aggression, which in turn was correlated with stereotypic behaviour. These results suggest delayed weaning as a simple means to improve the quality of life in domestic cats. Given that the cat is one of the most popular pets^34^, understanding the genetic and environmental factors that affect the welfare of cats is important. A high prevalence of behavioural issues was observed in our study cohort, indicating a need for improving welfare.

We utilised a citizen science approach by involving thousands of cat owners and breeders in the data collection, and collected a large behavioural data of home-living cats in multiple weaning age groups. However, the questionnaire approach has a few limitations. Questionnaires have a subjective component, but the reliability of questionnaires has been good in previous studies and the answers have strongly correlated with the behaviour of the animals^44–46^. Furthermore, sometimes questionnaires can detect some aspects of behaviour other methods may not detect^47^. As we used an online questionnaire, our behavioural data is a convenience sample. Enthusiastic cat people and people using social media were, therefore, more likely to respond to the questionnaire. However, this is not expected to affect our results, but this effect is likely seen in the high proportion of pedigree cats and low proportion of early weaned cats. Finally, our study is cross-sectional, rather than experimental. Therefore, based on our results, we cannot conclude that early weaning is the cause of the behaviour changes seen in this study. However, our results are in agreement with previous experimental studies on cats^39, 40^ and other animals^1, 7, 12^.

We discovered that early weaning increased the probability for aggression. In earlier studies conducted on laboratory cats, early weaned cats have showed elevated aggression towards both people and other cats^39, 40^. However, these cats were separated from their mothers much younger than separation usually occurs in companion cats. Furthermore, in one study, no differences in aggression were found between cats weaned at 6 weeks of age and at 12 weeks of age^40^. Moreover, the home environment is more environmentally and socially enriched than laboratory settings, which could reduce^48^ or even reverse some effects of early life stress^49^. However, abnormal aggression caused by severe early life stress seems to be quite resilient to enrichment^50^, which could explain why early weaned cats in our study show elevated aggression towards strangers even in a home environment.

Our results showed that delayed weaning decreased the probability of stereotypic behaviour. Supporting our findings, it has been previously noted that some forms of stereotypic behaviour are nursing behaviours that would normally be directed towards the mother^51^. Similarly, wool sucking in cats is thought to arise from the motivation to suckle^52^. A link between stereotypic wool sucking and early weaning was discovered in an earlier study^41^. Other studies have reported a similar effect in other animals, with early weaning increasing stereotypic behaviour motivated by nursing^53^ as well as general stereotypic behaviour^31, 32, 54^. Stereotypic behaviour was also affected by personality in our study. Cats scoring high in aggression and shyness displayed more stereotypic behaviour, as discovered earlier in rhesus macaques^29, 55^. Interestingly, the effect of weaning age on stereotypic behaviour decreased when the personality factors were included in the models. As weaning age influenced aggression as well, this indicates that weaning age affects stereotypic behaviour partially indirectly by influencing levels of aggression which, in turn, influences the occurrence of stereotypic behaviour. Furthermore, owners were more likely to report a behaviour problem in cats weaned before 8 weeks of age, indicating that subjectively cat owners regard the behaviour of these early weaned cats as problematic. The difference in the probability of behaviour problem between cats weaned before 8 weeks of age and between 12–13 weeks of age was large (probabilities being 18% and 7.9%, respectively), indicating that either early weaned cats differ from late weaned cats in some other behavioural traits than the ones studied here, or that our questionnaire does not accurately reflect the severity of the negative behavioural traits studied.

Based on our results and previous direct neurobiological studies in other species, early weaning may cause changes in brain function. We hypothesise that the behavioural changes in early weaned cats may be caused by dysfunction of cortical-basal ganglia circuits^56^. Socially deprived animals display decreased behavioural extinction as well as increased perseveration and behavioural inflexibility^9, 40^, and the same has been observed in stereotypic^57–59^ and aggressive^57^ animals. Neurobiologically, socially deprived animals have been discovered, for example, to be more sensitive to dopamine agonists^56, 60^ and have higher dopamine and lower serotonin levels in nucleus accumbens^56^, indicating changes in basal ganglia function. This hypothesis could be investigated further with, for example, non-invasive extinction tests^59^, a dopamine antagonist trial, or future metabolomics tools.

Early weaning may threaten the welfare of cats. Aggression is often defensive in nature or, in other words, induced by fear^61^. Furthermore, aggression arises in the hypothalamus and induces the activation of the HPA-axis^62^ and dopaminergic system^63^, which are also involved in stress. Therefore, it is likely that aggressive cats suffer from stress, either acute or chronic in nature. Stress can, in turn, impair health^64–67^. Furthermore, the occurrence of stereotypic behaviour can also threaten welfare. Cats that groom excessively may pull out patches of hair, leading to wounds that can become infected^68^. Similarly, symptoms of wool sucking include sucking and chewing textiles and plastics. Cats may swallow pieces of these materials, which can lead to intestinal blockages and even premature death. Moreover, aggression is a common reason for relinquishment in cats^69^ and aggressive cats may also be more likely to be euthanized. We unexpectedly discovered that cats weaned in adulthood or not weaned at all had a lower probability for aggression, shyness, and abnormal behaviour than other weaning age groups. Even more surprisingly, cats weaned at 14–15 weeks of age had a lower probability for excessive grooming than cats weaned at 12–13 weeks of age. Cat behaviour was previously thought to be relatively stable after the critical period of socialisation, which ends at 8 weeks of age^36^. We hypothesise that extended maternal care is the cause of this decrease in aggressive and abnormal behaviour, as many mothers may nurse and care for their kittens well into adulthood.

In the future, we plan to collect more data and extend our questionnaire to include more environmental factors. Many early life environmental factors, such as maternal care, living conditions before weaning, socialisation, and post-weaning living conditions, may affect behaviour and interact with weaning age, but as our questionnaire did not include these questions, these effects could not be addressed. Therefore, we aim to collect more data to study the interaction of weaning age with other environmental factors and to examine, whether breeds are differentially influenced by weaning age.

Our findings suggest that early weaning can lead to decreased mental welfare. As cats weaned at 14–15 weeks of age were at a lower risk for stereotypic behaviour, the welfare of home living cats may be improved by pushing the recommended weaning age to 14 weeks. This would be a simple and inexpensive way to improve companion feline welfare.

## Methods

### Questionnaire

A multiple choice online questionnaire was designed to collect extensive information on the health, living conditions and behaviour of Finnish domestic cats. Owners defined their cat’s activity level, tendency to seek human contact (later labelled as ‘contact with people’), aggressiveness towards family members, strangers, and other cats, as well as shyness towards strangers and novel stimuli, ranging from ‘not at all’ to ‘very much’ on a 5-point Likert-type scale. The owners also defined how much the cat licks, bites, and sucks itself using the same Likert-type scale. The owners were asked whether the cat bites, sucks, or eats wool or plastic (labelled as ‘wool sucking’). Here, the options were ‘never’, ‘1–3 times in the cat’s lifetime’, ‘1–12 times per year’, ‘1–4 times per month’, ‘1–3 times per week’, ‘daily’, ‘many times per day’, and ‘most of the day’. Furthermore, the owners were asked whether they thought that their cat had a behaviour problem with the answering options being ‘no’, ‘yes, self-evaluated’, and ‘yes, diagnosed by a veterinarian’. This subjective question helped us understand, which behaviours owners may find problematic. The questionnaire also included demographic questions, such as age, sex, breed, current presence of other cats in the household, and access to outdoors. Furthermore, cat owners could report the weaning age of the cat using nine categorical options: before 8 weeks of age, at 8–9 weeks of age, at 10–11 weeks of age, at 12–13 weeks of age, at 14–15 weeks of age, between 16 weeks and 1 year of age, in adulthood, not weaned at all, or weaning age unknown.

Informed consent was obtained from all participants. Cat owners were informed that the questionnaire answers would be used for research. We emphasized that all information given by the owners would remain strictly confidential and that individual cats or owners could not be identified from the published results.

The questionnaire was opened December 2012 and advertised on Facebook and via cat breed organisations. The questionnaire is still open and can be answered on the website http://www.kissangeenit.fi.

### Statistical analyses

Some new variables were created before analyses. Firstly, due to the large number of breeds with few individuals, some cat breeds were grouped together (Supplementary Table S1). These grouped breeds, such as Abyssinian and Somali, have a strong genetic relationship^70, 71^. Breeds with only a few respondents were combined under ‘other’ breed group. Secondly, non-intact cats were either castrated or spayed, had a hormonal implant inserted (males only), or were receiving contraceptive oral pills (females only); all of these methods inhibit fertility and change the hormonal status of the cat. Therefore, a new binomial variable called ‘hormonal status’ was created, in which cats were regarded as either intact or sterilized. The data initially consisted of 7397 cats. Cats with missing values in personality traits (N = 352) were removed. Furthermore, cats with missing information on weaning age (N = 60) or weaning age unknown (N = 936) were removed, as were cats with missing values in other explanatory variables (N = 302). After careful data examination, some cats had a clearly false age and they were removed as well (N = 21). At this point, the data consisted of 5726 individuals (4925 in wool sucking due to omitting the mildest wool sucking cases, 5683 individuals in grooming and 5550 in behaviour problem due to missing data).

Personality factors were used as covariates in the analyses, as behavioural traits may be intercorrelated. Therefore, we wanted to control for these variables, to understand whether early weaning impacts behavioural traits indirectly, by increasing another trait affecting the studied trait, rather than directly. To obtain these personality factors, principal component analysis was utilised. As the personality traits were coded on a Likert-type scale, polychoric correlations instead of Pearson correlations were used in the principal component analysis. Number of factors to be extracted was based on Kaiser’s stopping rule, scree test, and parallel analysis. Kaiser’s stopping rule suggested a three-factor solution, with eigenvalues of factors 3 and 4 being 1.11 and 0.62, respectively. Scree plot and parallel analysis confirmed this. Since the intercorrelations of the factors were low, orthogonal Varimax rotation was requested. The first factor consisted of all aggression traits and was labelled as ‘Aggression’, and the second factor consisted of shyness traits and was therefore labelled ‘Shyness’. The third factor was composed of activity level and contact with people and was hence dubbed ‘Extraversion’ (Supplementary Table S2).

Logistic regression was used to study the effects of early weaning on behaviour. Nine response variables (and therefore nine analyses) were used: behaviour problem; contact with people; aggression towards family members, strangers, and other cats; shyness towards novel objects and strangers; wool sucking; and excessive grooming. All the response variables were reduced to binomial categories. In all aggression and shyness traits, the event was levels 2–5. In grooming, the event consisted of levels 4–5, since some owners considered 1 as a normal level of grooming, whereas some considered the moderate level (3) as normal. In contact with people, low levels of contact were considered interesting and therefore the event consisted of levels 1–3, as most cats were reported to have a high level of contact to people. In wool sucking, ‘never’ was considered a non-event and occurrence of wool sucking of ‘1–4 times per month’ or higher constituted the event, as we wanted to compare the cats that never display wool sucking to cats that have displayed this behaviour frequently. In behaviour problem, the owner and veterinary diagnoses were grouped together and considered an occurrence of owner-evaluated behaviour problem.

Explanatory variables were selected based on previous literature and forward stepwise model selection by AIC values was used. The model selection was initiated with a starter model of weaning age, sex, and age, as weaning age was the variable of primary interest and different age groups and sexes tend to behave differently^72–76^. The model selection process favoured the inclusion of several additional variables in the model. In the tested models, we also included some interactions between the weaning age and other variables (Supplementary Table S3), but these interactions were not included in the final models as they decreased model fit. The final models and AIC model selection are shown in Supplementary Table S3.

Since early weaning was compared to the recommended weaning age of 12 weeks, contrasts between the early weaned categories (weaned under 8 weeks of age, at 8–9 weeks of age, and at 10–11 weeks of age) and the category weaned at 12–13 weeks of age were requested, as well as a contrast between early weaned cats and cats weaned at 14–15 weeks of age. Furthermore, a contrast between cats weaned in adulthood or not weaned at all and all the other cats, as well as a contrast between cats weaned at 12–13 weeks of age and 14–15 weeks of age were requested. As the number of pairwise comparisons was high due to several categorical variables, all P values, including the overall effect of variables, were controlled for false discovery rate to decrease the probability of type I error. Contrasts between the differentially weaned groups described above were chosen *a priori*, and thus the P values of these contrasts were not adjusted. Significance cut-off P value was set to P < 0.05.

Multicollinearity was tested by requesting the generalised variance inflation factor. All the variables fulfilled this assumption, with the variance inflation factor being under 2.5 in all the variables. Furthermore, Pearson goodness-of-fit test for each analysis was examined and all models had a non-significant P value. All statistical analyses were conducted using SAS software version 9.4 (SAS Institute Inc. 2002–2014), except for multicollinearity testing which was done with package car^77^ in R^78^.

### Data Availability

Data is available from the corresponding author on request, as the data is from privately owned family cats, and therefore there is a possibility to identify an individual cat from the data.

## Electronic supplementary material

Supplementary information
