# Supplementary material for: Early weaning increases aggression and stereotypic behaviour in cats
Source: Sci Rep. 2017 Sep 4;7:10412. doi: 10.1038/s41598-017-11173-5 (PMC5583233; doi:10.1038/s41598-017-11173-5)
Supplement: Supplementary file 1 — Supplementary information [file 41598_2017_11173_MOESM1_ESM.pdf]

Early weaning increases aggression and stereotypic behaviour in cats

Milla K. Ahola, Katariina Vapalahti, Hannes Lohi

Supplementary Table S1. Descriptive statistics.

		N	%
Sex	Female	3086	53.89
	Male	2640	46.11
Weaning age	<8 weeks	177	3.09
	8-9 weeks	223	3.89
	10-11 weeks	311	5.43
	12-13 weeks	2732	47.71
	14-15 weeks	1010	17.64
	16 weeks - 1 year	577	10.08
	As an adult	119	2.08
	Not weaned	577	10.08
Other cats in the household	No	874	15.26
	Yes	4852	84.74
Access to outdoors	Daily	1493	26.07
	3-5 times a week	767	13.4
	1-2 times a week	538	9.4
	1-2 times a month	577	10.08
	Few times a year	1280	22.35
	Never	1071	18.7
Neutering status	Intact	1261	22.02
	Neutered	4465	77.98

Cat breed group	Cat breed	N	%
ABY	Abyssinian	108	1.89
	Ocicat	180	3.14
	Somali	105	1.83
BEN	Bengal	228	3.98
BRI	British Shorthair	244	4.26
BUR	Burmese	242	4.23
	Burmilla	35	0.61
CRX	Cornish Rex	298	5.2
DRX	Devon Rex	213	3.72
EUR	European Shorthair	185	3.23
HCS	House Cat	836	14.6
KOR	Korat	174	3.04
MCO	Maine Coon	320	5.59
NFO	Norwegian Forest Cat	301	5.26
ORI	Balinese	41	0.72
	Oriental Longhair	24	0.42
	Oriental Shorthair	136	2.38
	Seychellois Longhair	2	0.03
	Seychellois Shorthair	7	0.12
	Siamese	97	1.69
PER	Exotic Shorthair	32	0.56
	Persian	194	3.39

RAG	Ragdoll	308	5.38
RUS	Russian Blue	268	4.68
SBI	Birman	293	5.12
SIB	Neva Masquerade	7	0.12
	Siberian	291	5.08
TUV	Turkish Angora	43	0.75
	Turkish Van	126	2.2
other	American Curl Longhair	56	0.98
	American Curl Shorthair	23	0.4
	American Shorthair	1	0.02
	Chartreux	17	0.3
	Cymric	33	0.58
	Don Sphynx	13	0.23
	Kurilian Bobtail	11	0.19
	Manx	56	0.98
	Egyptian Mau	13	0.23
	Sphynx	134	2.34
	Selkirk Rex	4	0.07
	other	27	0.47

Supplementary Table S2. Component loadings of the personality traits on three Varimax-rotated principal components.

Trait labels	Aggressiveness	Shyness	Extraversion
Aggression towards other cats	**0.75**	0.12	0.01
Aggression towards family members	**0.90**	0.08	-0.03
Aggression towards strangers	**0.88**	0.15	-0.05
Activity level	-0.02	-0.02	**0.89**
Shyness towards novel objects	0.15	**0.93**	-0.10
Shyness towards strangers	0.15	**0.93**	-0.17
Contact to people	-0.03	-0.26	**0.83**

Supplementary Table S3. The AIC model selection and the final models of logistic regression analyses. N = 5726 (personality trait analyses), N = 4925 (wool sucking analysis), N = 5683 (excessive grooming analysis), N = 5550 (owner-evaluated behaviour problem analysis).

Contact to people

Model	AIC	Shyness added	Breed added	Other cats added	Hormonal status added
Basic model (weaning age, sex, and age)	6194.818				
Shyness	**5935.28**	5935.28			
Breed	6089.582	**5819.472**	5819.472		
Other cats	6193.983	5932.355	**5813.898**	5813.898	
Hormonal status	6195.819	5926.21	5816.637	**5811.226**	**5811.226**
Access to outdoors	6196.279	5936.464	5820.469	5815.569	5813.516
Weaning age*other cats	6197.873	5938.217	5819.205	5819.205	5816.856
Aggressiveness	6191.126	5936.794	5821.452	5815.637	5812.857

Final model: Weaning age, sex, age, shyness, breed, other cats, hormonal status

Aggression towards family members

Model	AIC	Breed added	Other cats added	Hormonal status added	Extraversion added
Basic model (weaning, sex, and age)	3099.964				
Breed	**3015.063**	3015.063			
Other cats	3035.787	**2955.62**	2955.62		
Hormonal status	3092.051	3010.607	**2951.281**	2951.281	
Extraversion	3101.216	3014.387	2952.785	**2948.436**	**2948.436**
Access to outdoors	3100.015	3016.276	2958.094	2954.04	2951.08
Shyness	3101.86	3015.258	2955.328	2950.076	2947.642
Weaning age*other cats	3041.735	2959.611	2959.611	2955.212	2952.715

Final model: Weaning age, sex, age, breed, other cats, hormonal status, extraversion

Aggression towards strangers

Model	AIC	Breed added	Access to outdoors added	Other cats added	Hormonal status added	Shyness added
Basic model (weaning, sex, and age)	3362.713					
Breed	**3279.463**	3279.463				
Access to outdoors	3344.691	**3262.459**	3262.459			
Other cats	3345.145	3262.632	**3246.819**	3246.819		
Hormonal status	3353.584	3274.099	3257.595	**3241.758**	3241.758	
Shyness	3354.83	3275.327	3257.859	3242.744	**3238.925**	**3238.925**
Extraversion	3364.436	3279.418	3262.381	3245.887	3240.873	3237.654
Weaning age*other cats	3356.185	3274.414	3258.863	3258.863	3253.775	3250.789

Final model: weaning age, sex, age, breed, access to outdoors, other cats, hormonal status, shyness

**Aggression towards other cats**	AIC	Breed added	Hormonal status added	Other cats added	Shyness added	Access to outdoors added
Basic model (weaning, sex, and age)	7542.074					
Breed	**7444.595**	7444.595				
Hormonal status	7474.514	**7392.165**	7392.165			
Other cats	7498.481	7405.784	**7354.568**	7354.568		
Shyness	7490.371	7408.984	7365.922	**7329.202**	7329.202	
Access to outdoors	7524.745	7423.756	7374.277	7338.132	**7312.915**	**7312.915**
Extraversion	7534.58	7442.118	7389.526	7353.254	7328.414	7312.498
Weaning age*other cats	7509.741	7417.397	7365.875	7365.875	7340.067	7323.724

Final model: weaning age, sex, age, breed, hormonal status, other cats, shyness, access to outdoors

**Shyness towards novel objects**	AIC	Breed added	Hormonal status added	Extraversion added	Access to outdoors added	Other cats added
Basic model (weaning, sex, and age)	7372.532					
Breed	**7209.506**	7209.506				
Hormonal status	7287.195	**7142.620**	7142.620			
Extraversion	7335.832	7170.651	**7103.453**	7103.453		
Access to outdoors	7359.735	7187.721	7125.314	**7086.588**	7086.588	
Other cats	7357.747	7200.273	7134.165	7092.579	**7075.810**	**7075.8100**
Weaning age *other cats	7368.509	7212.386	7145.71	7103.944	7087.079	7087.079
Weaning age*sex	7375.219	7208.943	7141.172	7101.272	7084.300	7074.045
Aggressiveness	7369.085	7209.321	7143.389	7103.473	7087.104	7077.243

Final model: weaning age, sex, age, breed, hormonal status, extraversion, access to outdoors, other cats

**Shyness towards strangers**	AIC	Breed added	Extraversion added	Hormonal status added	Other cats added
Basic model (weaning, sex, and age)	7873.925				
Breed	**7678.998**	7678.998			
Extraversion	7791.883	**7603.068**	7603.068		
Hormonal status	7817.186	7631.347	**7555.133**	7555.133	
Other cats	7869.034	7677.789	7599.844	**7552.338**	**7552.338**
Access to outdoors	7875.907	7675.616	7599.46	7554.362	7551.62
Weaning age*other cats	7872.085	7683.372	7605.44	7557.477	7557.477
Weaning age*sex	7878.784	7682.201	7605.81	7557.164	7554.589
Aggressiveness	7870.500	7677.009	7599.347	7552.752	7551.039

Final model: weaning age, sex, age, breed, extraversion, hormonal status, other cats

**Wool sucking**	AIC	Breed added	Access to outdoors added	Shyness added	Extraversion added	Aggressive-ness added
Basic model (weaning, sex, and age)	4978.367					
Breed	**4883.292**	4883.292				

Access to outdoors	4937.595	**4835.361**	4835.361			
Shyness	4937.384	4849.526	**4801.842**	4801.842		
Extraversion	4954.324	4867.687	4818.304	**4786.709**	4786.709	
Aggression	4962.224	4874.703	4827.48	4788.315	**4774.684**	**4774.684**
Other cats	4979.382	4884.94	4837.016	4803.595	4788.644	4776.629
Weaning age*other cats	4988.618	4988.618	4948.223	4907.807	4886.961	4868.731

Final model: weaning age, sex, age, breed, access to outdoors, shyness, extraversion, aggressiveness

Excessive grooming

	AIC	Breed added	Shyness added	Aggressiveness added	Access to outdoors added	Extraversion added
Basic model (weaning, sex, and age)	2343.552					
Breed	**2305.749**	2305.749				
Shyness	2314.938	**2282.797**	2282.797			
Aggression	2326.239	2289.818	**2260.844**	2260.844		
Access to outdoors	2327.743	2296.641	2273.322	**2251.815**	2251.815	
Extraversion	2332.362	2301.285	2279.433	2258.187	**2248.728**	**2248.728**
Other cats	2339.043	2300.291	2277.618	2259.039	2249.884	2247.297
Weaning age*other cats	2338.281	quasi-complete separation of data points				

Final model: weaning age, sex, age, breed, shyness, aggressiveness, access to outdoors, extraversion

Owner-evaluated behaviour problem

	AIC	Aggression added	Stereotypy added	Shyness added	Breed added	Other cats added
Basic model (weaning, sex, and age)	3305.6					
Aggression	**3190.595**					
Stereotypy	3246.079	**3143.281**	3143.281			
Shyness	3277.504	3148.98	**3108.287**	3108.287		
Breed	3292.842	3181.491	3135.417	**3097.588**	3097.588	
Other cats	3307.369	3188.503	3139.986	3104.747	**3095.031**	**3095.031**
Access to outdoors	3313.247	3197.885	3150.544	3116.386	3105.829	3103.24
Extraversion	3307.6	3192.304	3143.281	3108.558	3095.662	3093.661

Final model: weaning age, sex, age, aggression, stereotypy, shyness, breed, other cats

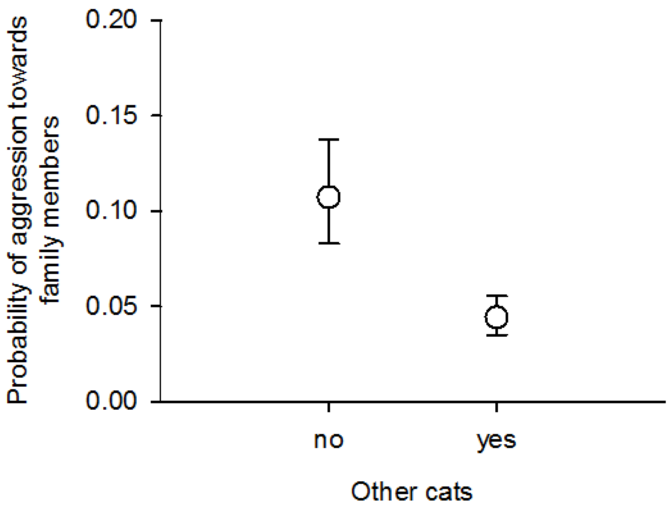

Supplementary Figure S1. Effect of the presence of other cats in the household on the probability of aggression towards family members. N = 5726. Error bars indicate 95% confidence limits.

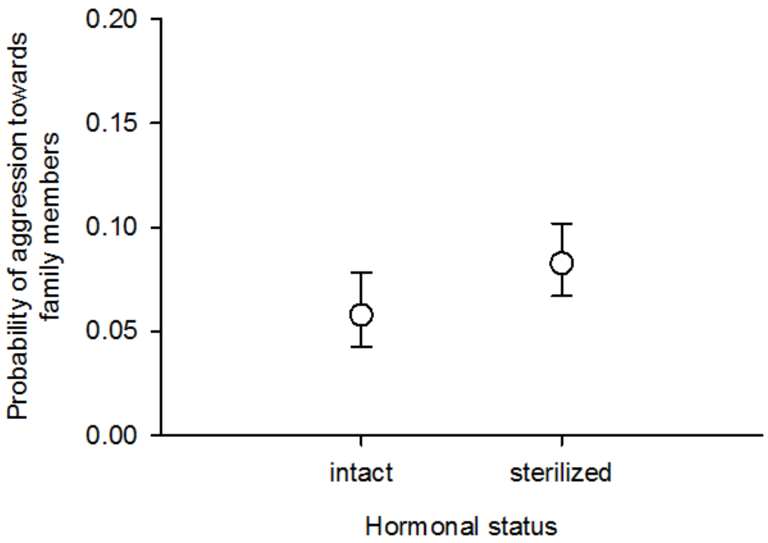

Supplementary Figure S2. Effect of hormonal status on the probability of aggression towards family members. N = 5726. Error bars indicate 95% confidence limits.

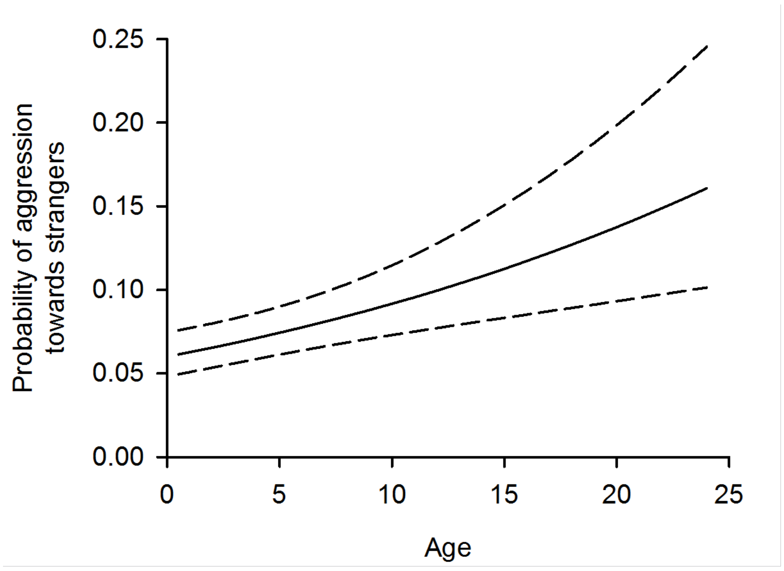

Supplementary Figure S3. Effect of age on the probability of aggression towards strangers. N = 5726.

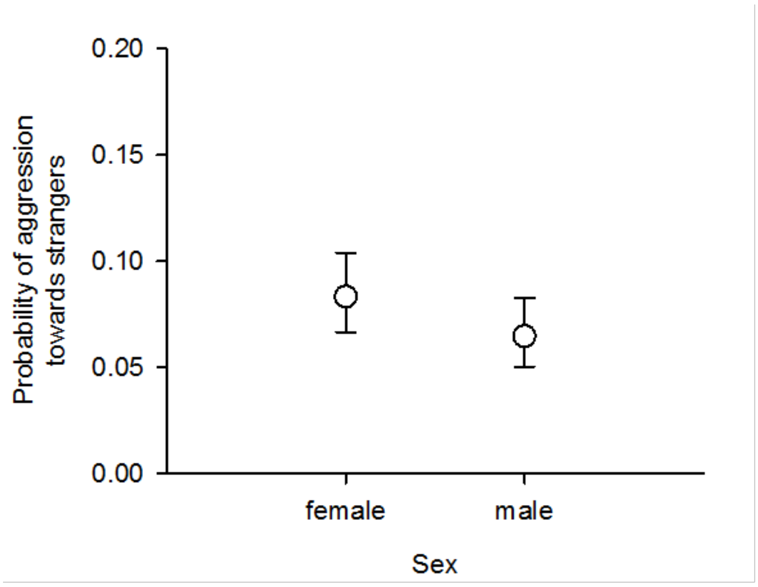

Supplementary Figure S4. Effect of sex on the probability of aggression towards strangers. N = 5726. Error bars indicate 95% confidence limits.

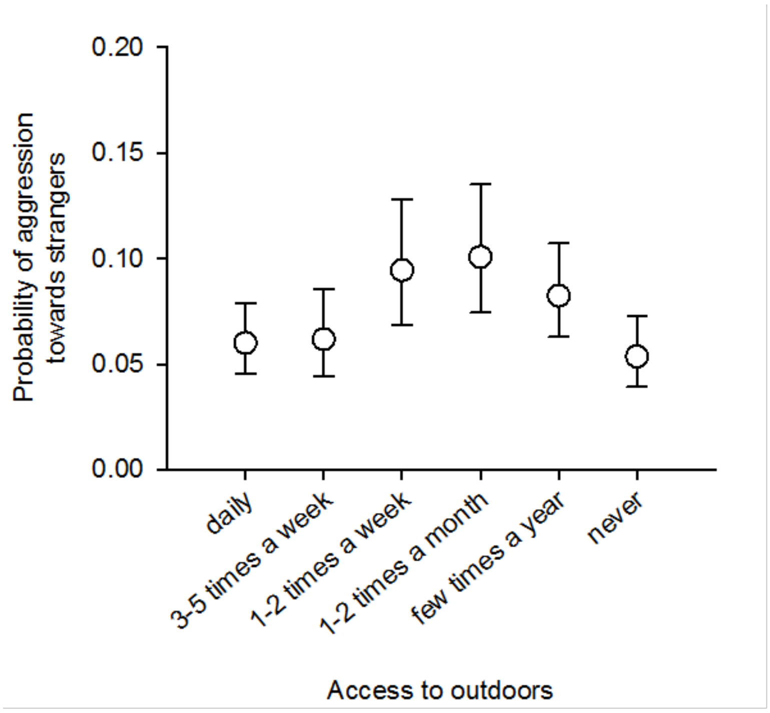

Supplementary Figure S5. Effect of access to outdoors on the probability of aggression towards strangers. N = 5726. Error bars indicate 95% confidence limits.

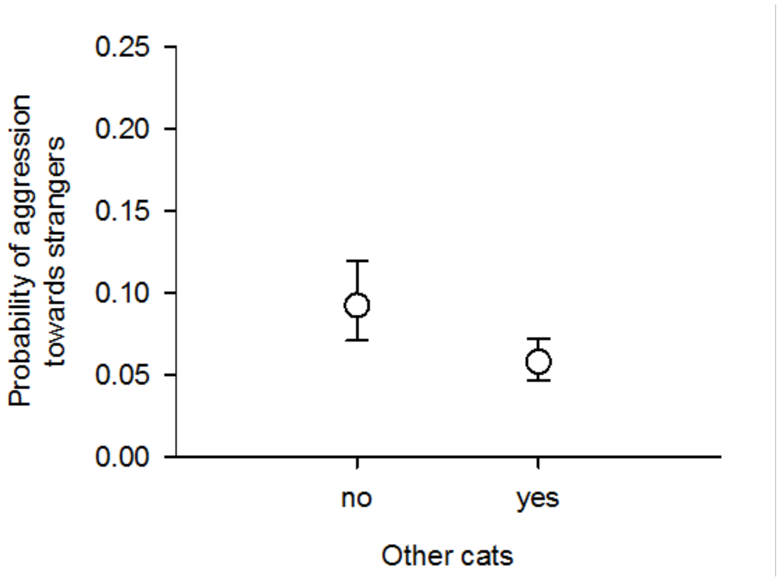

Supplementary Figure S6. Effect of the presence of other cats in the household on the probability of aggression towards strangers. N = 5726. Error bars indicate 95% confidence limits.

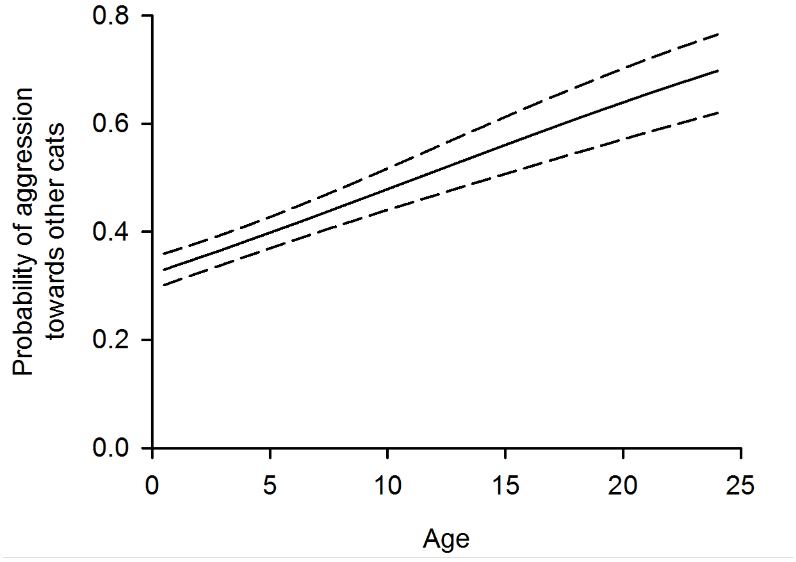

Supplementary Figure S7. Effect of age on the probability of aggression towards other cats. N = 5726.

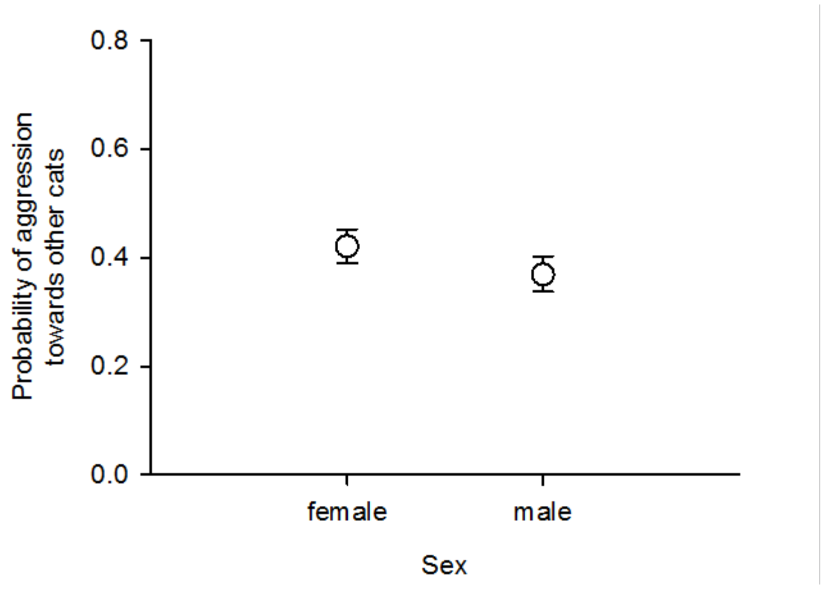

Supplementary Figure S8. Effect of sex on the probability of aggression towards other cats. N = 5726. Error bars indicate 95% confidence limits.

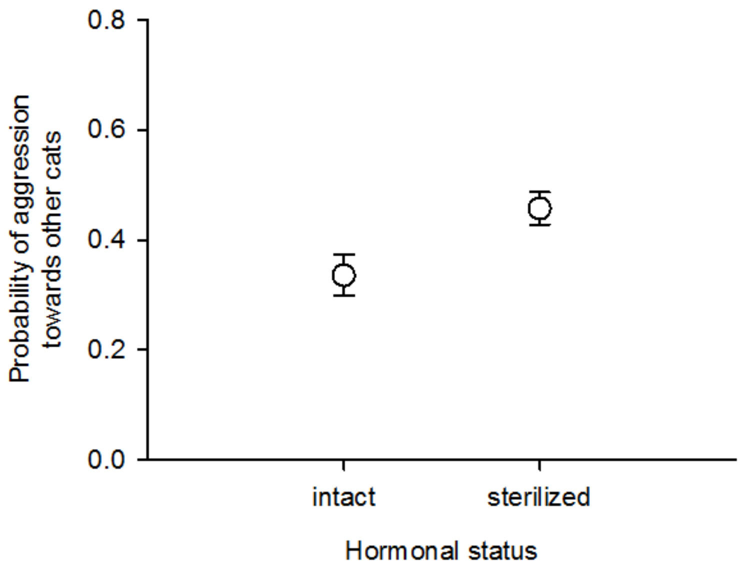

Supplementary Figure S9. Effect of hormonal status on the probability of aggression towards other cats. N = 5726. Error bars indicate 95% confidence limits.

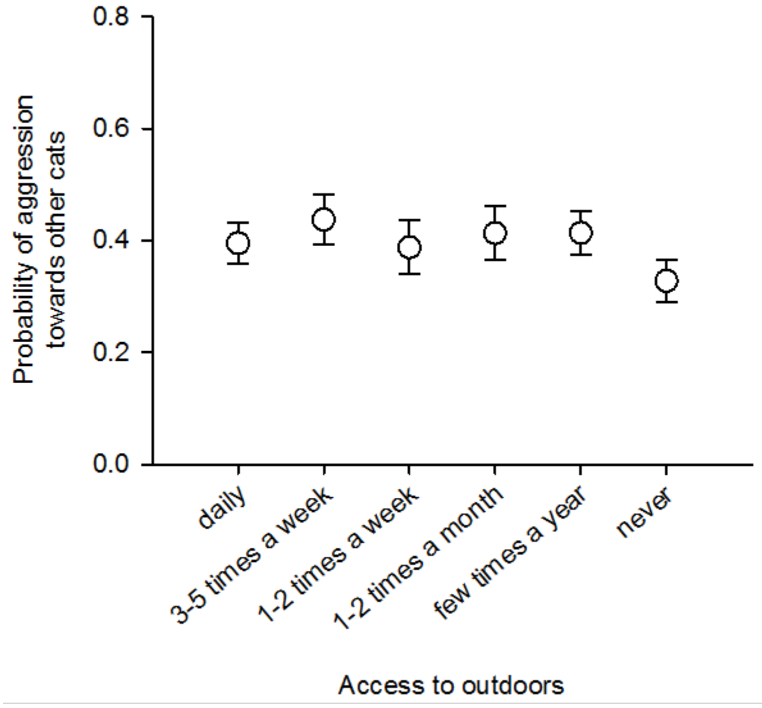

Supplementary Figure S10. Effect of access to outdoors on the probability of aggression towards other cats. N = 5726. Error bars indicate 95% confidence limits.

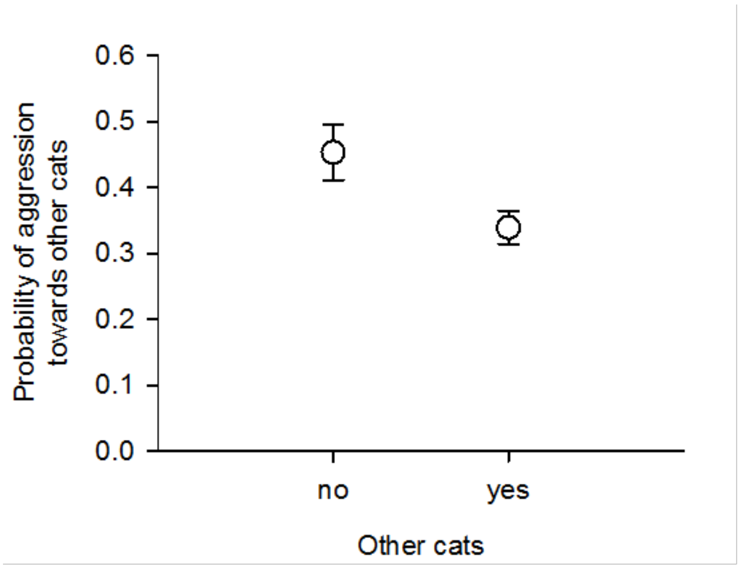

Supplementary Figure S11. Effect of the presence of other cats in the household on the probability of aggression towards other cats. N = 5726. Error bars indicate 95% confidence limits.

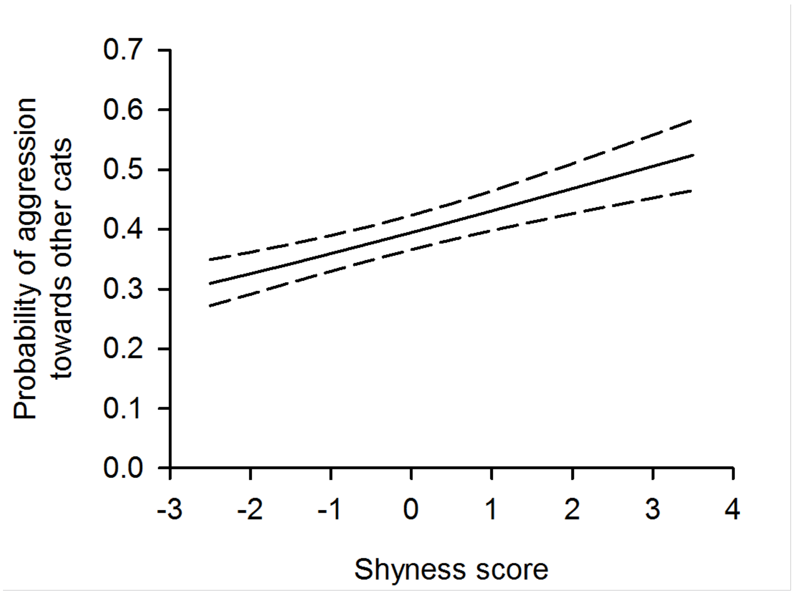

Supplementary Figure S12. Effect of shyness score on the probability of aggression towards other cats. N = 5726.

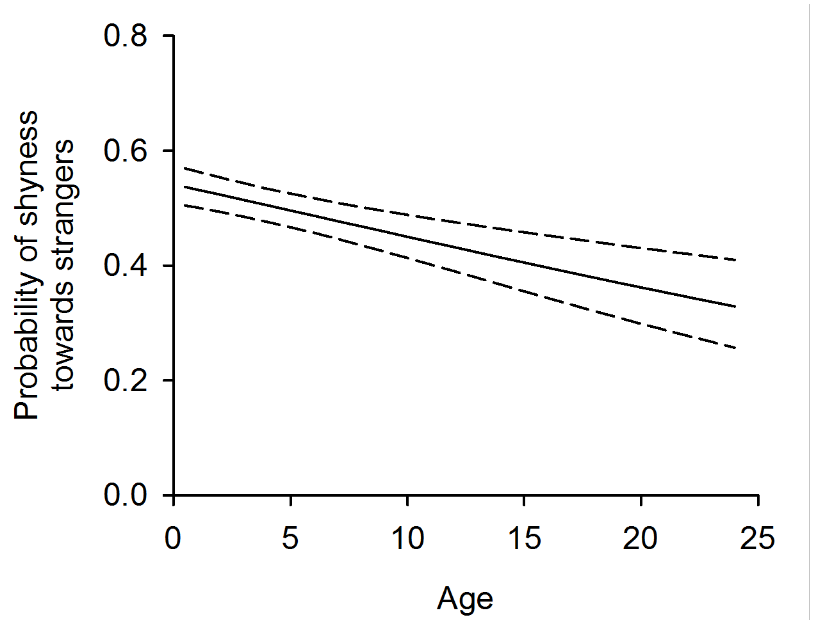

Supplementary Figure S13. Effect of age on the probability of shyness towards strangers. N = 5726.

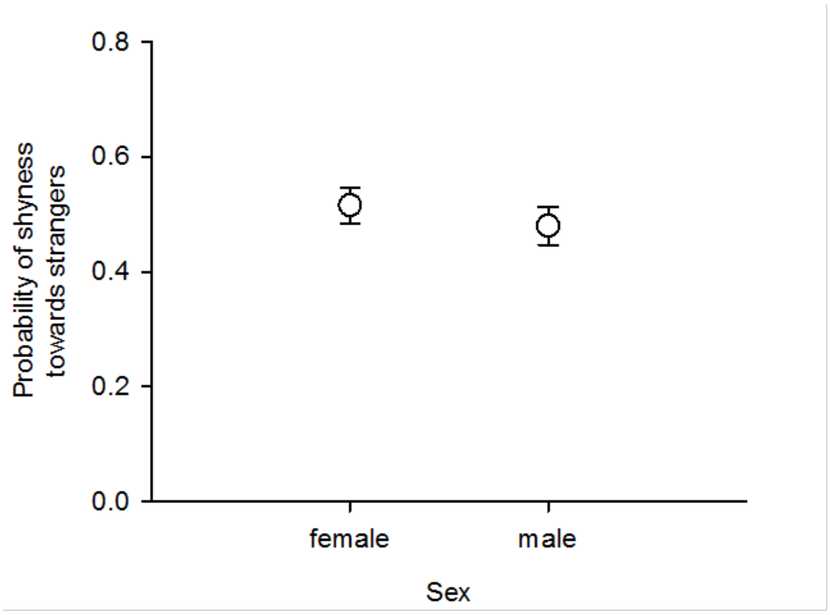

Supplementary Figure S14. Effect of sex on the probability of shyness towards strangers. N = 5726. Error bars indicate 95% confidence limits.

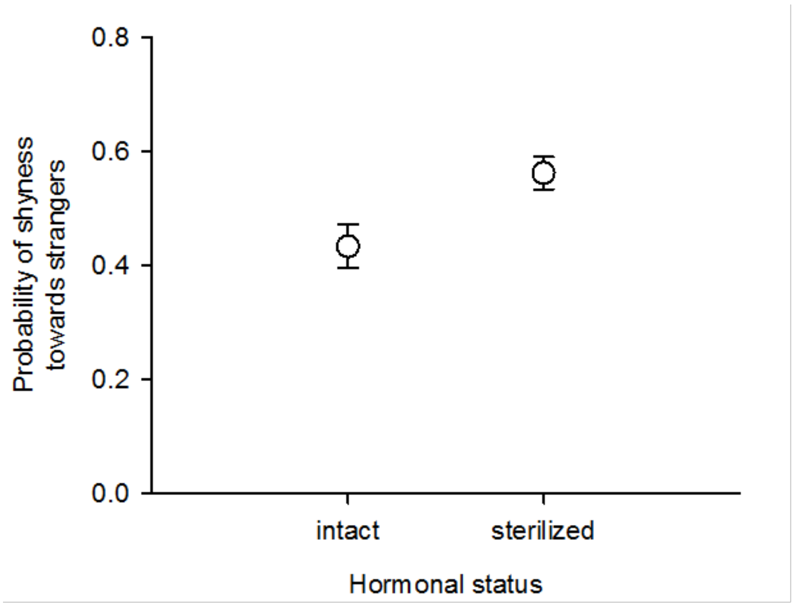

Supplementary Figure S15. Effect of hormonal status on the probability of shyness towards strangers. N = 5726. Error bars indicate 95% confidence limits.

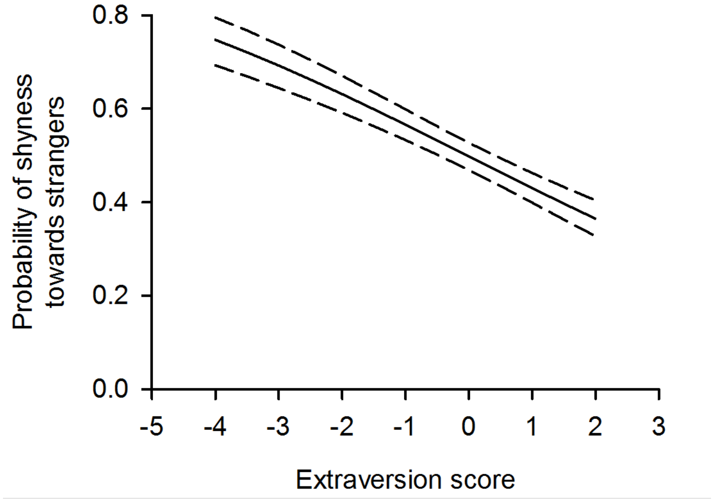

Supplementary Figure S16. Effect of extraversion score on the probability of shyness towards strangers. N = 5726. Error bars indicate 95% confidence limits.

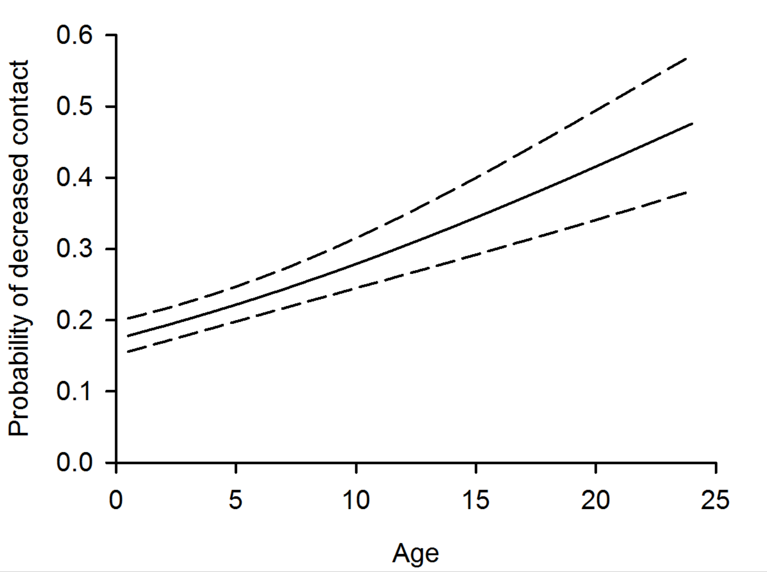

Supplementary Figure S17. Effect of age on the probability of decreased contact. N = 5726.

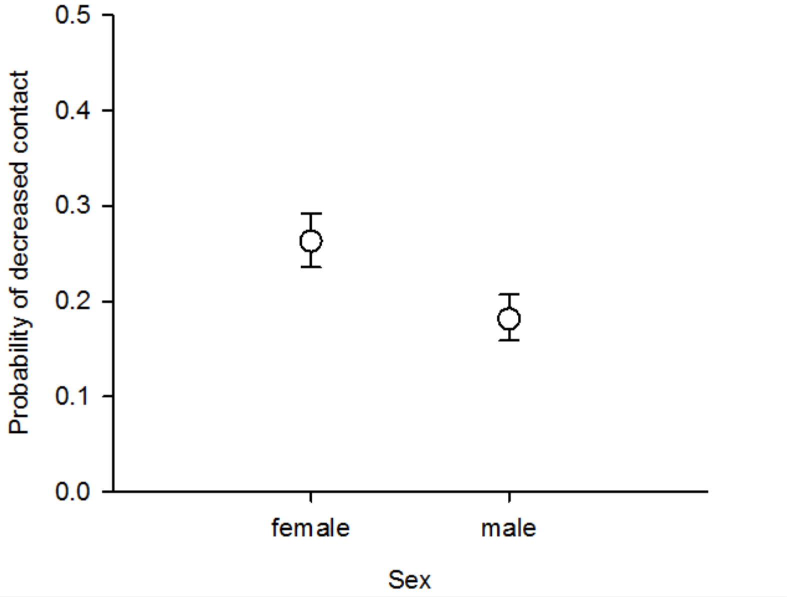

Supplementary Figure S18. Effect of sex on the probability of decreased contact. N = 5726. Error bars indicate 95% confidence limits.

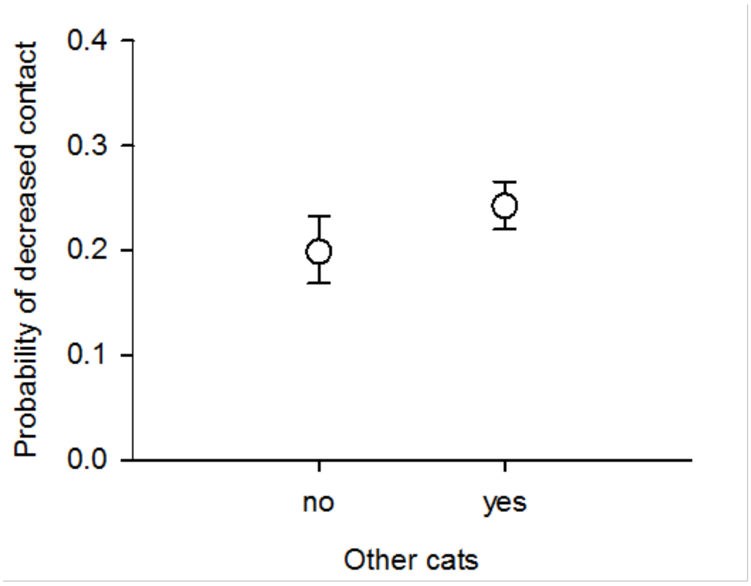

Supplementary Figure S19. Effect of the presence of other cats in the household on the probability of decreased contact. N = 5726. Error bars indicate 95% confidence limits.

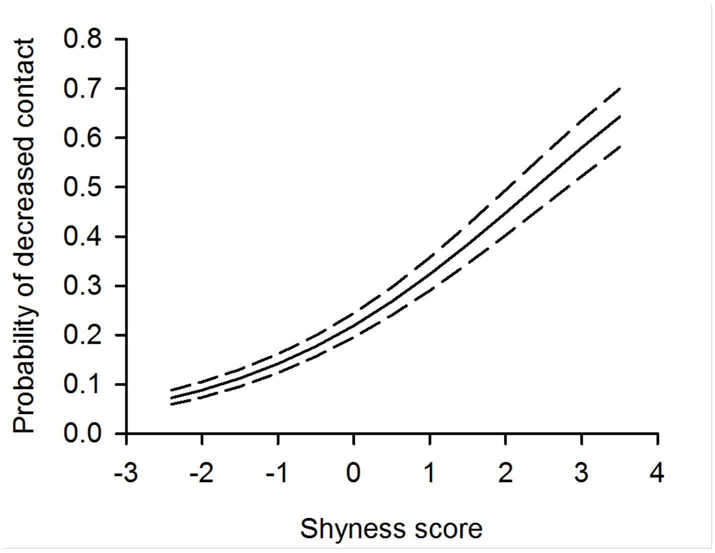

Supplementary Figure S20. Effect of shyness score on the probability of decreased contact. N = 5726.

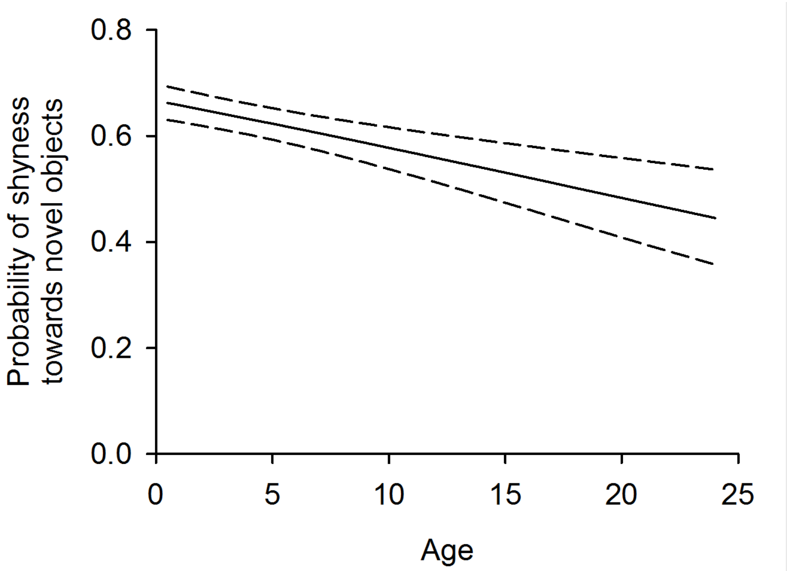

Supplementary Figure S21. Effect of age on the probability of shyness towards novel objects. N = 5726.

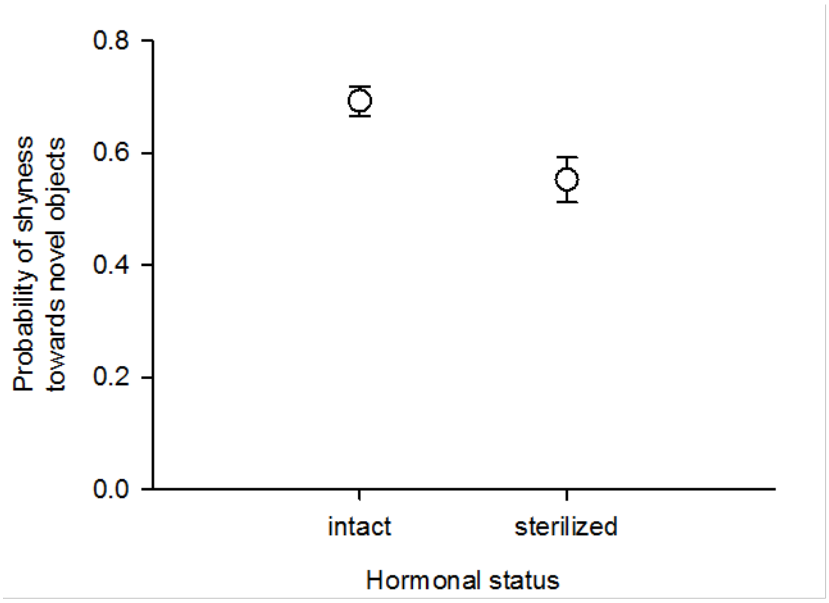

Supplementary Figure S22. Effect of hormonal status on the probability of shyness towards novel objects. N = 5726. Error bars indicate 95% confidence limits.

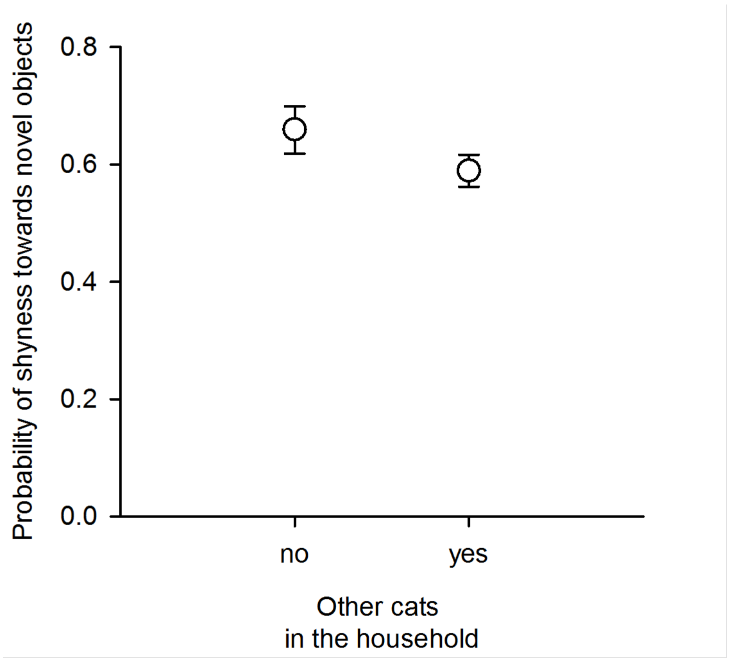

Supplementary Figure S23. Effect of the presence of other cats in the household on the probability of shyness towards novel objects. N = 5726. Error bars indicate 95% confidence limits.

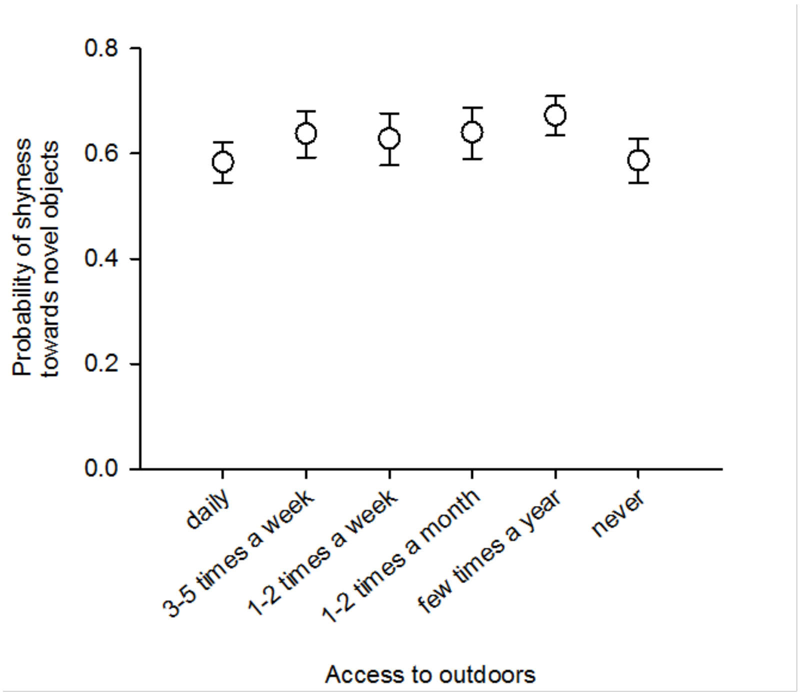

Supplementary Figure S24. Effect of access to outdoors on the probability of shyness towards novel objects. N = 5726. Error bars indicate 95% confidence limits.

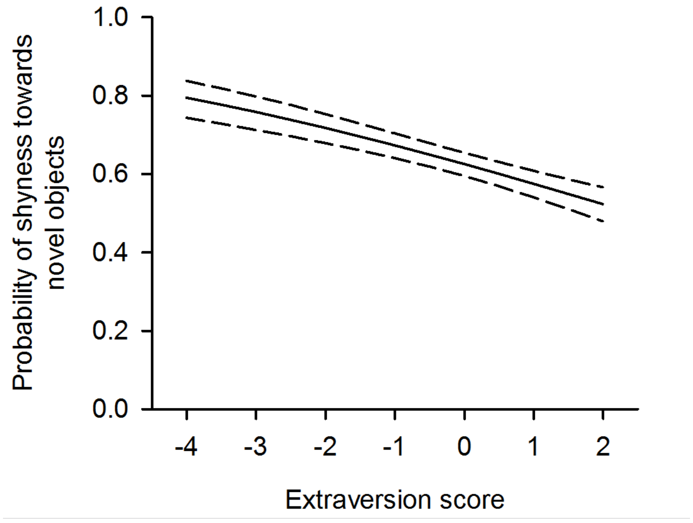

Supplementary Figure S25. Effect of extraversion score on the probability of shyness towards novel objects. N = 5726.

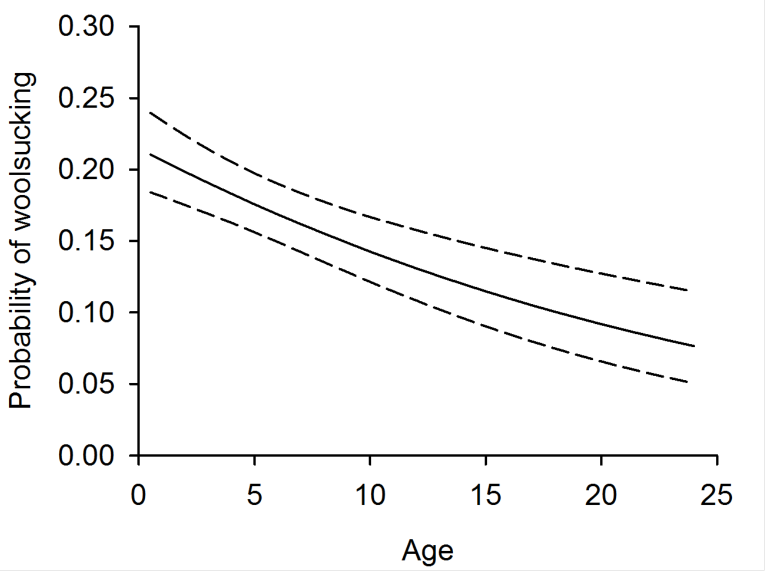

Supplementary Figure S26. Effect of age on the probability of wool sucking. N = 4925.

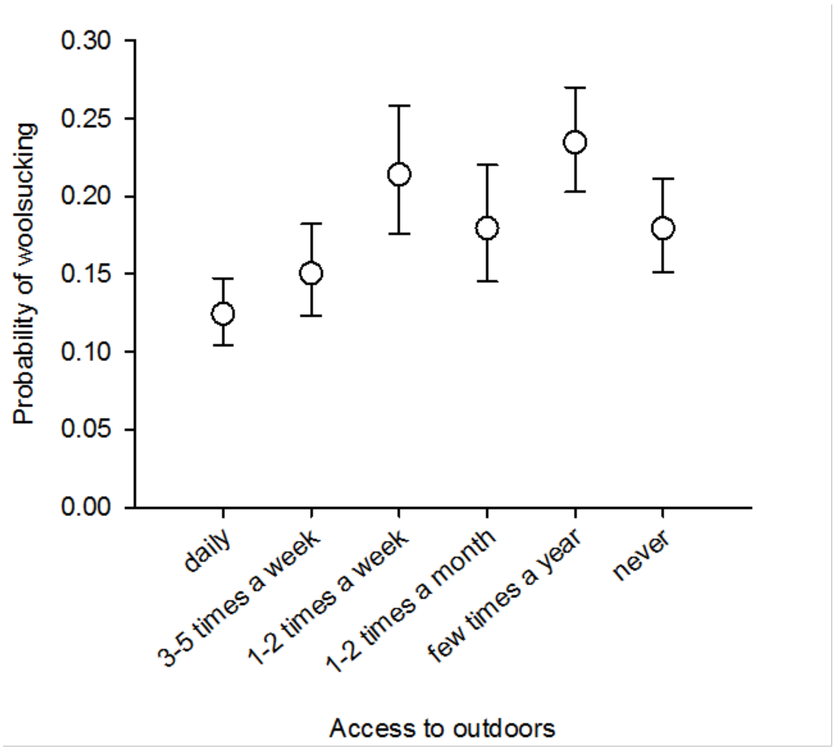

Supplementary Figure S27. Effect of access to outdoors on the probability of wool sucking. N = 4925. Error bars indicate 95% confidence limits.

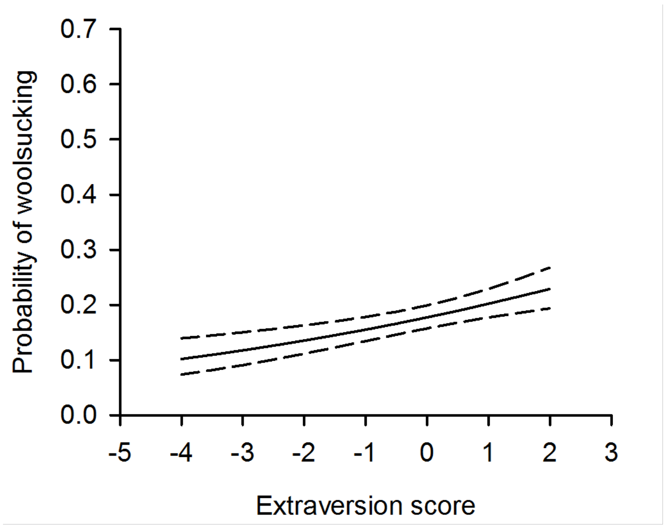

Supplementary Figure S28. Effect of extraversion score on the probability of wool sucking. N = 4925.

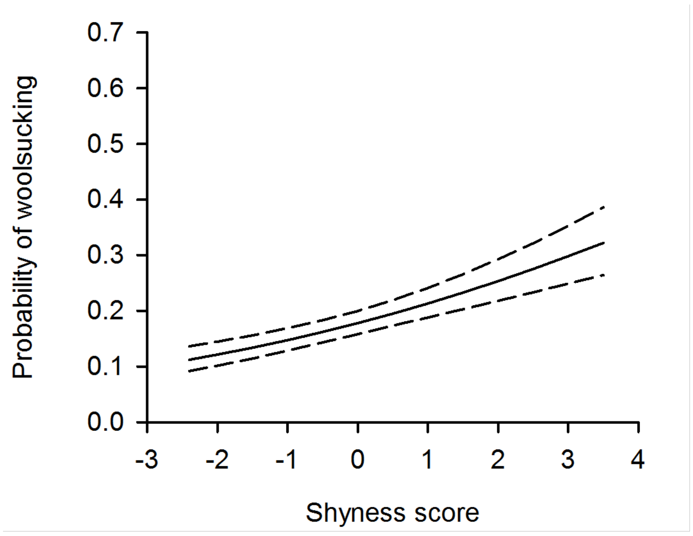

Supplementary Figure S29. Effect of shyness score on the probability of wool sucking. N = 4925.

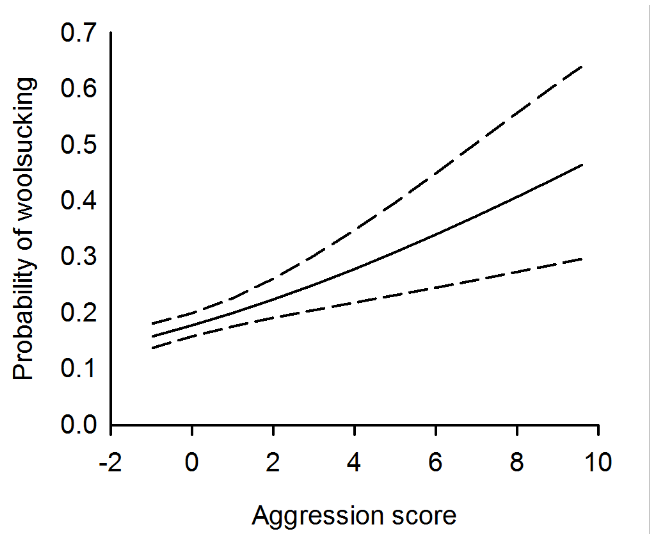

Supplementary Figure S30. Effect of aggression score on the probability of wool sucking. N = 4925.

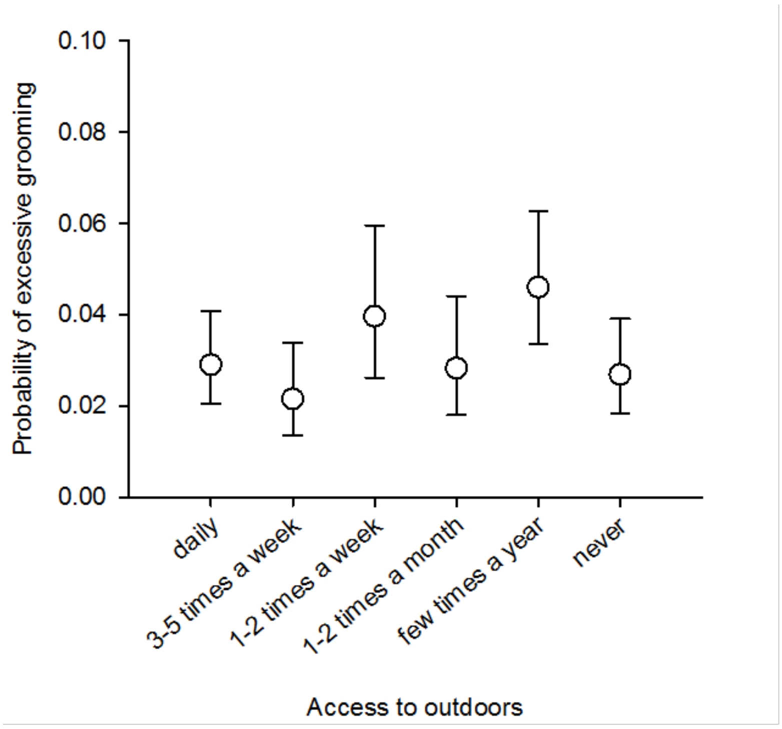

Supplementary Figure S31. Effect of access to outdoors on the probability of excessive grooming. N = 5683. Error bars indicate 95% confidence limits.

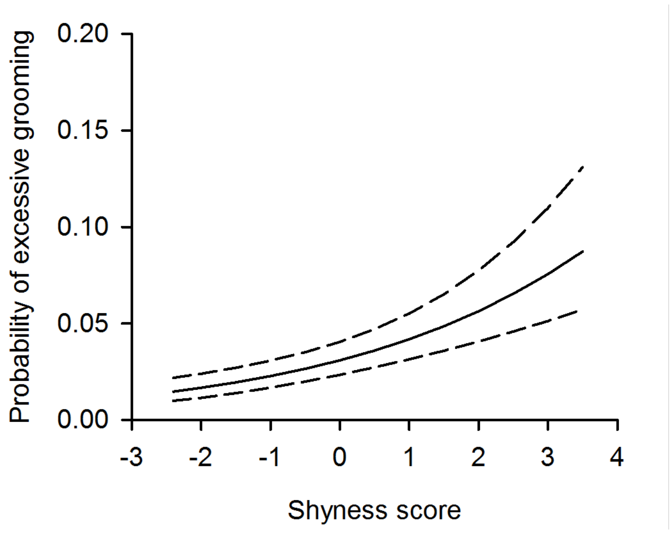

Supplementary Figure S32. Effect of shyness score on the probability of excessive grooming. N = 5683.

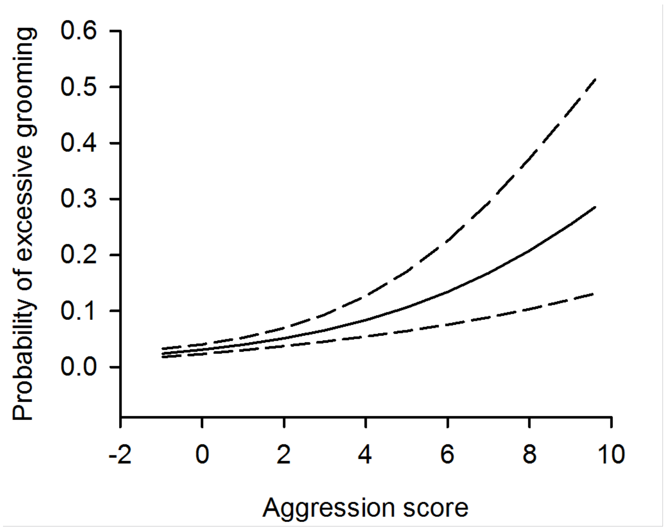

Supplementary Figure S33. Effect of aggression score on the probability of excessive grooming. N = 5683.

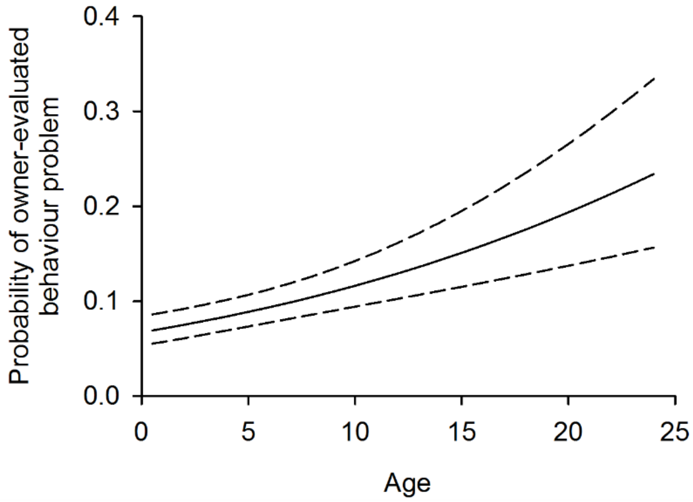

Supplementary Figure S34. Effect of age on the probability of owner-evaluated behaviour problem. N = 5550.

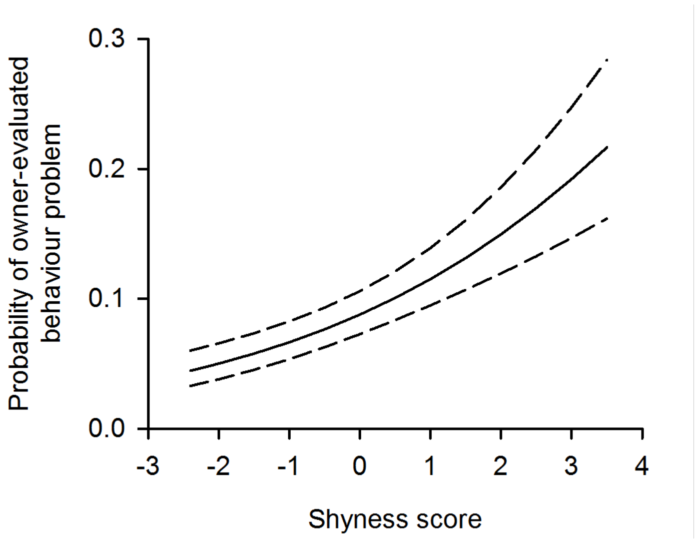

Supplementary Figure S35. Effect of shyness score on the probability of owner-evaluated behaviour problem. N = 5550.

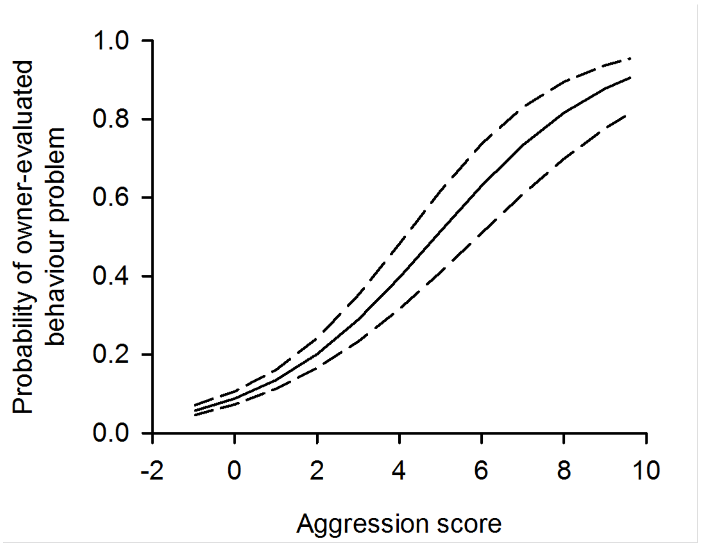

Supplementary Figure S36. Effect of aggression score on the probability of owner-evaluated behaviour problem. N = 5550.

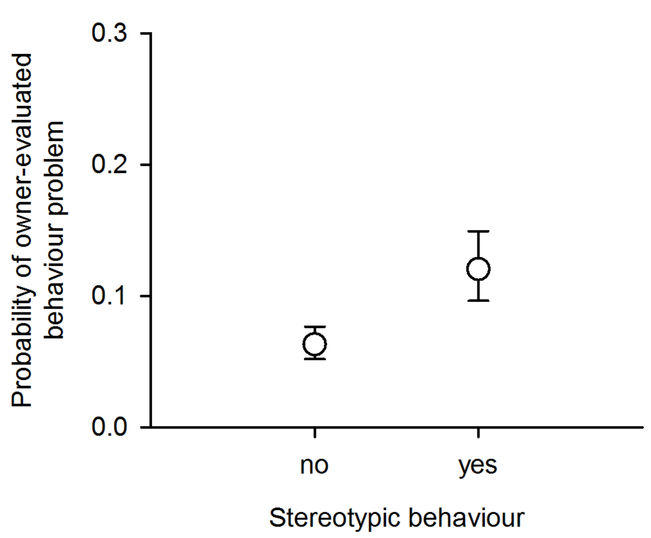

Supplementary Figure S37. Effect of the occurrence of stereotypic behaviour on the probability of owner-evaluated behaviour problem. N = 5550.
